# Ruminal utilization of nitrogen derived from ^15^N-labeled faba beans in vitro

**DOI:** 10.3168/jdsc.2025-0791

**Published:** 2025-08-20

**Authors:** S.E. Räisänen, Þ.H. Sigurðardóttir, A. Halmemies-Beauchet-Filleau, O. Pitkänen, A. Honkanen, T. Kokkonen, F.L. Stoddard, A. Simojoki, E. Sahlstedt, K.T. Rinne-Garmston, A. Vanhatalo

**Affiliations:** 1 Department of Agricultural Sciences, University of Helsinki, FI-00014 Helsinki, Finland; 2 Stable Isotope Laboratory of Luke (SILL), Natural Resources Institute Finland (Luke), FI-00790 Helsinki, Finland

## Abstract

The aim of this short-term in vitro study was to characterize and evaluate ruminal utilization of N fractions of faba bean protein. Faba bean seeds were grown using a ^15^N-enriched N-fertilizer and fractionated into insoluble N (INSN), NDIN, NPN, and soluble protein N (SPN). Unfractionated ground ^15^N-labeled faba bean seeds (FB) were used as a reference treatment. Batches of the N fractions and FB were incubated for 10 h in an automatic Gas Endeavor in vitro batch-culture system with 3 replicated incubations. Purified mono-, di-, and polysaccharides were used as an energy source. The ratio of buffer to rumen fluid, obtained from 2 rumen-cannulated cows, was 2:1. The incubation medium was sampled at 0, 0.5, 1, 2, 5, and 10 h after the start of incubation. Subsequently, the incubation medium was separated into low- (i.e., feed and protozoa) and high-speed centrifugate (i.e., bacterial) N pools and ammonia-N pool, and ^15^N enrichment of each pool was measured. Due to low gas and VFA production, data for NPN fraction were not included in the dataset. The faba bean seeds contained (% of CP) 54% SPN, 18% NPN, 17% NDIN, 10% true protein soluble in neutral detergent, and 2% ADIN. At the end of incubation, the recovery of ^15^N in the ammonia-N pool was greatest for FB (12%) and SPN (7%) compared with the INSN (2%) and NDIN (0.06%) fractions. The recovery of ^15^N in the bacterial-N pool was numerically greatest for the FB treatment (15%), followed by INSN and SPN (6%), and was lowest for the NDIN treatment (0.75%). In line with our hypothesis, ruminal metabolism of N differed between the N fractions of faba bean being greatest for the SPN fraction, and minimal for the NDIN. Future studies should confirm the findings from this short-term in vitro batch-culture study, and further investigate the degradation and ruminal utilization of faba bean soluble N and its contribution to MP supply in dairy cows fed diets with varying protein and energy contents.

## Highlights

• High rumen degradability of faba bean often results in low nitrogen efficiency.
• Majority of faba bean protein is soluble, with the potential to contribute to metabolizable protein supply.
• Soluble protein is degraded to NH3 and partly incorporated into bacterial nitrogen.
• The flow of soluble faba bean protein into the lower gut remains to be investigated.

## Graphical Abstract

**Figure:**
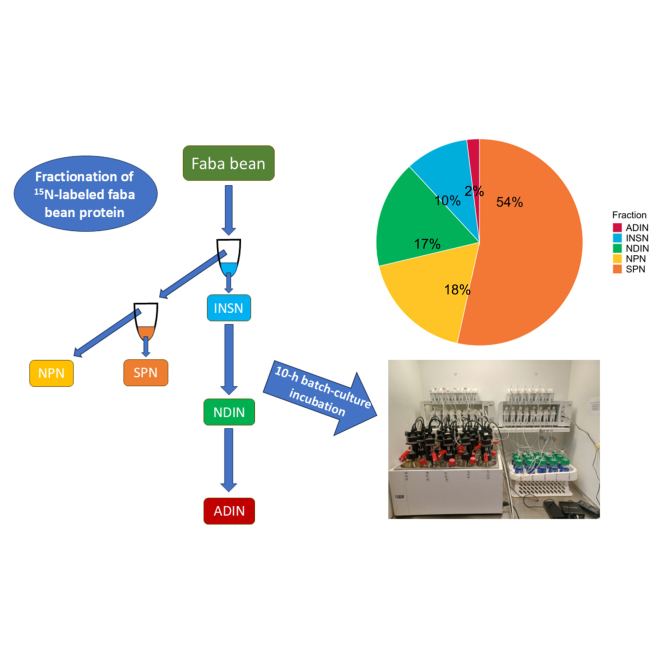
**Summary:** This study aimed to investigate ruminal metabolism of ^15^N-enriched faba bean protein and its nitrogen fractions (acid detergent insoluble N [ADIN], insoluble N [INSN], neutral detergent insoluble N [NDIN], nonprotein N [NPN], and soluble protein N [SPN]). The results confirmed the high content of soluble true protein nitrogen in faba bean protein. The 10-hour batch culture incubation of ^15^N-enriched faba bean protein showed that it was largely degraded to ammonia (12%) and partly incorporated into bacterial nitrogen (15%). Future studies should further evaluate the degradation rate and utilization of soluble faba bean protein in the rumen and its contribution to postruminal amino acid supply and nitrogen utilization.

Faba bean (*Vicia faba* L.) seeds have been investigated for high CP and starch contents as an alternative protein feed to substitute rapeseed meal or cake (Puhakka et al., 2016; Lamminen et al., 2019), or soybean meal (Cherif et al., 2018; Hansen et al., 2021) in dairy cow diets. However, production responses to faba bean have varied from equal (Hansen et al., 2021, Pitkänen et al., 2023) to reduced responses (e.g., Lamminen et al., 2019; Räisänen et al., 2023) in comparison to conventional protein feeds. This has often been attributed to the estimated high amount of RDP in faba bean relying on in situ determination of rumen degradation kinetics (Hansen et al., 2021; Räisänen et al., 2023). Feed evaluation systems include various ways to describe protein fractions in feed for estimation of ruminal degradation and its effects on microbial protein synthesis, which contribute to predictions of MP supply from a given ration. The in situ method has been widely used for estimation of ruminal protein degradability (e.g., Nozière, 2018; NASEM, 2021), but it cannot account for soluble protein N (**SPN**; Huhtanen et al., 2014) and is susceptible to errors such as microbial contamination, especially in starchy feeds (Varvikko and Lindberg, 1985). The Cornell Net Carbohydrate and Protein System (CNCPS) model (Sniffen et al., 1992a; Fox et al., 2004) describes feed proteins based on chemical fractionation of N, including SPN. However, Choi et al. (2002) and Ahvenjärvi et al. (2018) demonstrated that a significant portion of soluble NAN can flow out of the rumen, which has important implications for predicting MP supply from protein feeds with high soluble protein content, such as faba bean. Stable isotope-labeled N (^15^N) has been used as a marker to study in vitro ruminal kinetics and utilization of feed N, including whole feeds, soluble feed N (Ahvenjärvi et al., 2009), and several feed N fractions (e.g., Melgar and Hristov, 2004; Vaga and Huhtanen, 2018). This marker technique allows for tracking of the metabolism and utilization of N beyond chemical analysis, or indirect estimations based solely on factors such as ruminal ammonia concentration or the in situ method. Though the RDP fraction of faba bean is considered high, knowledge is limited on the ruminal utilization of its N fractions. Therefore, the objective of this short-term in vitro batch-culture study was to characterize N fractions of faba bean and to decipher the distributions between rumen N pools utilizing ^15^N-isotope labeling of faba bean protein. We hypothesized that faba bean protein, mainly consisting of SPN with its fermentation end product ammonia-N, would be available for rumen microbial protein synthesis, whereas its INSN and NDIN fractions would be used to a smaller degree.

Faba bean (cultivar ‘Sampo‘) was grown at the Viikki Research Farm (60° N, 25° E) and labeled by fertilization with ^15^N in the field as described in Itkonen et al. (2024). The whole biomass of faba bean was harvested at maturity and dried in a cold-air drier for 2 d, whereafter the seeds were further dried at 30°C to 40°C in a forced-air oven for 1 d, and ground to pass through a 1-mm sieve. Chemical composition presented in Table 1, including DM, OM, ash, CP, NDF, ADF, and starch, of the ground ^15^N-labeled faba bean seeds (**FB**) was determined using standard procedures described in Puhakka et al. (2016). The ^15^N content of the FB and each N fraction (see later description) was determined using an Elemental Analyzer (Europa EA GSL, Sercon Ltd., UK) coupled with an Isotope Ratio Mass spectrometer (20–22 IRMS, Sercon Ltd., UK).

**Table 1:** Chemical composition and N fractions of ^15^N-labeled faba beans used in the in vitro batch-culture incubations

Item	^15^N-labeled faba bean
DM, %	91.0
Chemical composition, % of DM	
OM	86.8
CP	32.0
NDF	14.6
ADF	13.1
Starch	33.6
Ash	4.17
N fraction,^1^ % of CP	
A	17.6
B_1_	54.1
B_2_	9.86
B_3_	16.7
C	1.66
EPD, %	79.2

1 Determined according to Licitra et al. (1996). A = NPN; B_1_ = SPN; B_2_ = true protein insoluble in mineral buffer but soluble in neutral detergent; B_3_ = NDIN; C = ADIN. Effective protein degradability (EPD) was calculated according to Fox et al. (2004) with degradation rates for B_1_, B_2_, and B_3_: 2, 0.1, and 0.002/h, respectively, and passage rate of 0.08/h.

The FB were further processed in 100-g batches to separate SPN, NPN, insoluble N (**INSN**), NDIN, and ADIN fractions, according to Licitra et al. (1996). The experimental treatments included SPN, INSN, and NDIN fractions as well as unfractionated ground FB as a reference treatment. The NPN fraction resulted in low total gas production and VFA concentrations, leading to its exclusion. The inclusion rate of each fraction was based on their N and ^15^N contents to ensure an equal amount of 1 mg of ^15^N per incubation bottle (due to the low N-content of NDIN, only 0.5 mg of ^15^N was included for this treatment) and was 0.668, 5.86, 13.3, and 1.67 g of DM for SPN, INSN, NDIN, and FB, respectively. The atom percent excess (**APE**; analyzed ^15^N atom percent corrected for background ^15^N abundance of 0.3663) was 1.26, 1.26, 0.997, and 1.24 for SPN, INSN, NDIN, and FB, respectively. Purified mono-, di-, and polysaccharides (i.e., 33 mg of dl-glucose [Sigma-Aldrich], 60 mg of maltose [TCI Europe], 33 mg of sucrose [Thermo Scientific], 242 mg of soluble corn starch [Sigma-Aldrich], and 495 mg of apple pectin [TCI Europe]) were used as energy sources for the incubation medium.

All experimental procedures involving cannulated cows were approved by the National Animal Experiment Board in Finland, as described by Räisänen et al. (2023). Rumen contents from 2 rumen-cannulated (11 cm silicone elastomer cannulas; Rumen Cannula, Mount Evelyn, Australia) lactating Nordic Red cows (milk yield of 30.2 and 34.9 kg/d, BW of 680 and 664 kg, DIM of 221 and 229 d, respectively) were collected and combined for the inoculum. Whole rumen contents were collected from 4 locations of the reticulorumen (the ventral sac, reticulum, and caudal and dorsal sections of the feed mat) and filtered through 2 layers of cheesecloth. The solid rumen contents were transferred into a container with warm McDougall's buffer (McDougall, 1948). The rumen fluid and solids were brought to the laboratory, and filtered separately under continuous flushing with CO_2_ through a sieving tower (smallest sieve size 0.25 mm). The rumen solids with the buffer were shaken vigorously for 45 s before filtering. Last, an equal volume of rumen fluid and rumen solids buffer was mixed to form the incubation medium, flushed with CO_2_, and placed in a 39°C water bath for start of incubation.

The incubation was conducted with a Gas Endeavor device (Bioprocess Control Sweden AB, Lund, Sweden) and included 6 incubation bottles (500 mL) per incubation (i.e., the 5 experimental treatments and a blank). The incubation lasted for 10 h and consisted of 3 replicate runs. To start the incubation, each incubation bottle was flushed with CO_2_ and 120 mL of rumen inoculum was added (total incubation medium volume 180 mL). The Gas Endeavor device recorded total gas and CH_4_ production continuously throughout the incubation; these data were used only for confirming active fermentation in bottles and are not presented.

Incubation medium samples of 20 mL were collected at 0 h (start of incubation), 0.5, 1, 2, 5, and 10 h under continuous flushing with CO_2_. The incubation medium samples were centrifuged at 500 × *g* for 10 min at 4°C. The low-speed centrifugate pellet (**LSC**) was freeze-dried, weighed, ground with a mortar and pestle, and analyzed for ^15^N enrichment in the LSC pool (protozoa and feed) as described previously. The supernatant was further centrifuged at 20,000 × *g* for 15 min at 4°C (repeated thrice), and the high-speed pellet (considered as the bacterial pool) was freeze-dried and analyzed for ^15^N enrichment in the bacterial-N pool. Subsamples of the supernatant were further processed for determination of ^15^N enrichment in the ammonia-N pool, and analysis of VFA and total ammonia-N concentrations. The sample processing for ^15^N-enriched ammonia-N analysis followed the standard protocol (Brooks et al., 1989; Hristov et al., 2001). To obtain the amount of ^15^N enrichment in each N pool, the weight of the freeze-dried pellets (LSC and bacterial fractions) was multiplied by their respective N concentration and the APE. For the ammonia pool, the concentration of ammonia-N measured in the incubation medium was used. The recovery of ^15^N in each pool was calculated as the ^15^N content of the pool divided by the amount of total ^15^N recovered, and multiplied by 100.

Data were analyzed using PROC MIXED of SAS (version 9.4; SAS Institute Inc., Cary, NC). The statistical model included the fixed effects of treatment and time (start and endpoint), their interaction, and the random effect of replicate. Time was used as a repeated term when all 6 sampling time points were included in the dataset, and spatial power [i.e., sp(pow)] was used as the covariance structure for the repeated term due to unequal spacing of sampling times. The distribution of ^15^N within the N pools was expressed as compositional data that were therefore subjected to center-log-ratio transformation and analyzed using the same model as described previously without the repeated term. Treatment comparisons were done using the Tukey-Kramer method within a time point. All data are presented as least squares means. Statistical differences were considered significant at *P* ≤ 0.05 and a trend at 0.05 < *P* ≤ 0.10.

This short-term incubation was conducted to evaluate faba bean protein fractions and their ruminal utilization, when provided as the sole N source. The low replicate number is noted and was due to the limited availability of the isotope-labeled faba bean. The 10-h incubation time was selected to characterize N utilization, but may have been too short for a full characterization of NDF-bound N dynamics in the rumen. The endpoint fermentation profile (data not shown) was within a normal range: pH of the incubation medium ranged from 5.51 for INSN to 6.55 for SPN (*P* < 0.01; SEM = 0.049), and total VFA averaged 101 m*M* (SEM = 9.34), acetate 65.8 mol-% (SEM = 0.71), propionate 18.4 mol-% (SEM = 0.58). These data serve as an initial step, providing insight into utilization of different protein fractions from faba bean, and the differential utilization of N fractions by rumen microbes.

The chemical composition of the unfractionated ^15^N-labeled faba bean and the distribution of N fractions in the faba bean protein are presented in Table 1. As expected based on our hypothesis, the majority of faba bean protein was B_1_ (i.e., SPN), followed by fractions A and B_3._ Around 10% of the protein was B_2_, and the smallest proportion was of fraction C. Effective protein degradability (**EPD**), calculated from the N fractions, was 79%. Distribution of N fractions in faba bean protein in the current experiment (cultivar 'Sampo') was similar to those reported for cultivar 'Kontu' by Räisänen et al. (2023) but differed from the values for cultivar ‘Kontu’ reported by Kuoppala et al. (2021): there were more of both fraction A (18% vs. 13% of CP) and fraction B_3_ (17% vs. 8% of CP) in the faba bean of current study. The calculated EPD, however, was similar (∼80%) between the studies. Thus, these data confirm the high content of SPN in faba bean protein (i.e., fraction B_1_; 54% to 62% of CP; current study, Räisänen et al. [2023], and Kuoppala et al. [2021]) compared with other commonly used protein feeds, such as rapeseed meal with a reported B_1_ fraction of 21%, 28%, and 10% of CP (Stefański et al., 2013; Kuoppala et al., 2021; Räisänen et al., 2023), or soybean meal with a reported B_1_ of 20% to 23% of CP (Sniffen et al., 1992; NASEM, 2021). It is important to note the high content of SPN in faba bean protein, as it may contribute more to the protein flowing out of the rumen than is accounted for in many of the current feed evaluation systems (Choi et al., 2002; Ahvenjärvi et al., 2018).

Distribution of ^15^N within the incubation medium N pools at the beginning and end of the incubation is presented in Figure 1. At the beginning of incubation, more than 95% of the ^15^N was present in the LSC pool (mostly feed), being greatest (*P* < 0.01) for NDIN and lowest for INSN. At the end of incubation, the recovery of ^15^N in the ammonia-N pool was greatest (*P* ≤ 0.03) for FB and SPN, followed by INSN, and was lowest for NDIN. The recovery of ^15^N in the bacterial-N pool followed numerically a similar sequence as the ammonia-N pool. Total recovery of ^15^N in the LSC pool was greatest (*P* < 0.02) for the NDIN fraction, followed by INSN, then SPN, and was lowest for the FB treatment. At the start of incubation, NDIN (*P* < 0.001; Figure 2A) had a lower ^15^N-enrichment in the ammonia-N pool compared with FB treatment, but did not differ from the other fractions, and NDIN treatment had a lower (*P* ≤ 0.009) ^15^N-enrichment level compared with all other treatments from 0.5 h until the end of incubation. Further, the FB treatment had a greater (*P* ≤ 0.004) enrichment level than INSN and SPN from 0.5 to 5 h of incubation, and at 10 h compared with SPN. At 5 h of incubation, INSN had a greater (*P* < 0.009) enrichment level in the ammonia-N pool compared with SPN, but did not differ at other times. The ^15^N-enrichment of the bacterial-N pool was greater for the FB treatment compared with all other treatments throughout the incubation (*P* ≤ 0.03; Figure 2B). Further, INSN had a greater (*P* = 0.004) enrichment level compared with NDIN at the beginning of the incubation, and greater than both NDIN and SPN at 5 and 10 h of incubation. Last, SPN had a greater (*P* < 0.001) enrichment level of the bacterial-N pool at 5 and 10 h of incubation compared with the NDIN.

**Figure 1:**
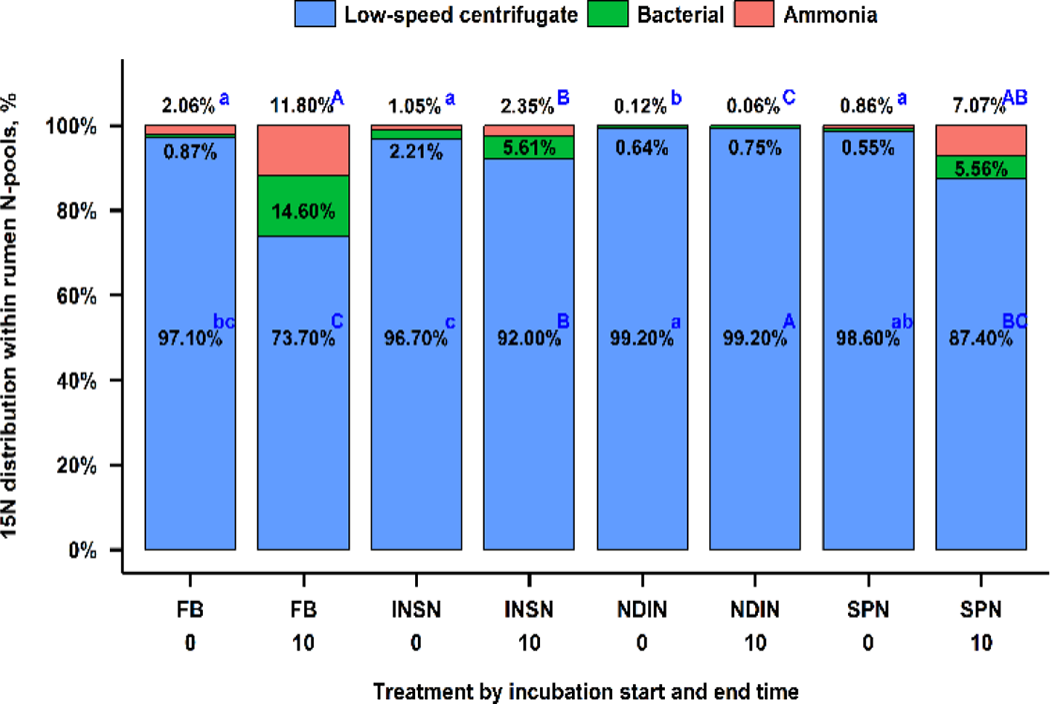
Distribution of ^15^N within the incubation medium N pools (ammonia, bacterial, and low-speed centrifugate) at the beginning and end of a 10-h in vitro batch-culture incubation (data in figure shown as untransformed values). Treatments are FB = unfractionated ground ^15^N-labeled faba bean seed; INSN = insoluble N fraction of FB; NDIN = NDF-bound N fraction; SPN = soluble protein N fraction. n = 24 (number of observations used in statistical analysis). SEM: 0.141, 0.167, and 0.157 (center log-ratio transformed data) for ammonia, bacterial, and low-speed centrifugate pools, respectively. Low-speed centrifugate includes feed and protozoa separated by centrifuging at 500 × *g* for 15 min at 4°C. Means with different lowercase letters (a–c) differ at *P* ≤ 0.05 within an N pool at 0 h. Means with different uppercase letters (A–C) differ at *P* ≤ 0.05 within an N pool at 10 h.

**Figure 2:**
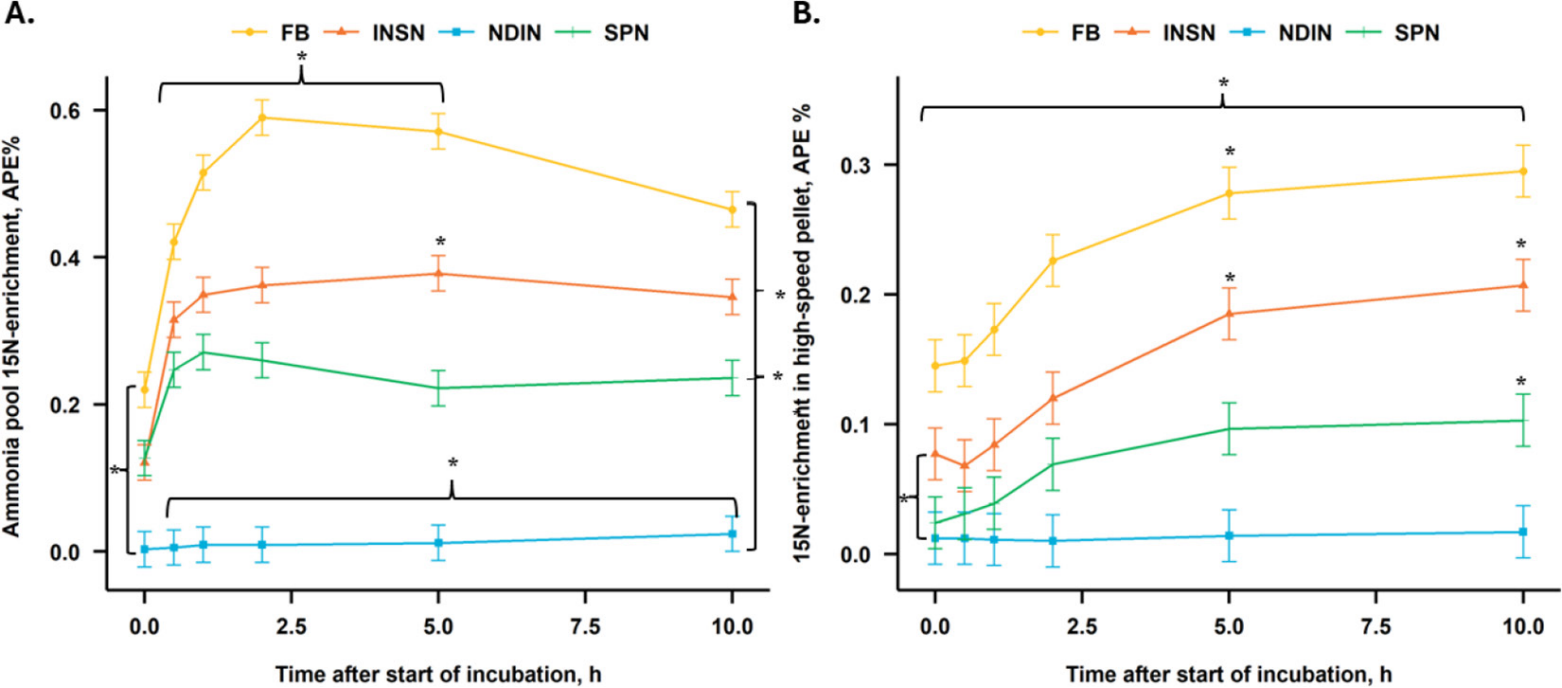
^15^N-enrichment in (A) ammonia and (B) bacterial N pools across a 10-h in vitro batch-culture incubation. Treatments are FB = unfractionated ground ^15^N-labeled faba bean seed; INSN = insoluble N fraction of FB; NDIN = NDF-bound N fraction; SPN = soluble protein N fraction. n = 72 (number of observations used in statistical analysis). SEM = 0.0244 for ammonia pool; SEM = 0.00963 for bacterial pool. Error bars indicate SEM. *P ≤ 0.05. Panel A: at 0 h FB versus NDIN; at 0.5, 1, 2, 5, and 10 h NDIN versus all other treatments; at 0.5, 1, 2, and 5 h FB versus INSN and SPN; at 5 h INSN versus SPN; at 10 h FB versus SPN. Panel B: all time points FB versus all others; 0 h INSN versus NDIN; at 5 and 10 h INSN versus SPN and NDIN; at 5 and 10 h SPN versus NDIN.

There was a clear shift of ^15^N into the ammonia-N pool for both the FB and SPN treatments from 0 to 10 h of incubation (from 2% to 12% and 0.86% to 7% of total ^15^N, respectively). A more moderate (from 1% to 2.4%) to no change (average of 0.15%) was observed in the ammonia-N pool for the INSN and NDIN, respectively. Interestingly, Vaga and Huhtanen (2018) reported a relatively high recovery of INSN fraction from ^15^N silage as ammonia-N at early stages of their 28-h in vitro incubation and indicated an availability of INSN for microbial protein synthesis. They further observed a greater uptake of ^15^N ammonia with the INSN fraction than with the SPN fraction, similar to an in vitro study by Peltekova and Broderick (1996) with a 6-h incubation investigating the degradation of SPN and INSN fractions from alfalfa hay and silage.

In the current study, the initial ammonia and bacterial ^15^N pools were greater for the INSN (1.1% and 2.2%) than for the SPN (0.86% and 0.55%) and NDIN fractions (0.12% and 0.65%). This might indicate a direct uptake of the INSN fraction by the rumen microbes as suggested for soluble and NDF-bound N by Vaga and Huhtanen (2018) and Stefański et al. (2020), respectively. At the end of incubation, the ^15^N distribution in the bacterial pool was similar for the INSN and SPN fractions (5.6%), whereas the ^15^N in the ammonia pool was relatively high for the SPN fraction. The lack of transfer of the ^15^N from the LSC pool of NDIN fraction to either the ammonia or bacterial pools indicates a very low availability of NDF-bound N for both direct bacterial uptake as speculated by Vaga and Huhtanen (2018), or its degradation to ammonia-N and uptake as such by rumen microbes as an N source.

The bacterial ^15^N pool increased numerically the most for the FB treatment (from 0.87% to 15% of total ^15^N), which can be expected as it provided a more complete profile of available N in the N-deficient conditions (i.e., a low ammonia-N concentration in the incubation medium). In addition, both starch (∼560 mg) and fiber (∼244 mg of NDF) as additional energy sources were available, likely improving the overall fermentation rates and N utilization. In the current experiment, the SPN fraction exhibited the largest distribution of ^15^N in the ammonia-N pool at the end of incubation of the 3 N fractions tested, which indicates a degradation and deamination of the SPN (a true protein source) by the rumen microbes. However, a notable portion (5.6%) of the ^15^N from the LSC with the SPN fraction was also transferred to the bacterial-N pool. This was likely a result of both uptake of the ammonia-N released from SPN as well as direct uptake of soluble true protein N into bacterial cells (Stefański et al., 2020). It is evident that in the first hour of the incubation, there was a rapid degradation of both the SPN and INSN into ammonia-N, whereafter it stabilized for the INSN, but decreased up to 5 h of incubation for the SPN fraction. Much of the ammonia-N released during the first hours of incubation from the ^15^N-labeled INSN and SPN fraction was then likely taken up by the microbial cells. This can be seen in the steady increase in the ^15^N enrichment of the bacterial pool for both the INSN and SPN fractions over the course of the 10-h incubation.

It is noted that in this in vitro batch-culture study, the lack of outflow of rumen contents may not have fully captured the potential contributions of faba bean soluble protein to postruminal MP supply, as has also been shown in vitro for grass silage and rapeseed meal soluble N (e.g., Ahvenjärvi et al., 2009, 2018; Stefański et al., 2020). However, animal feeding studies (e.g., Puhakka et al., 2016; Kuoppala et al., 2021) have shown an increased ruminal ammonia concentration or increased MUN indicating a degradation and deamination of untreated faba bean protein in the rumen, as indicated also in the current in vitro study. Nevertheless, faba bean also has a potential to enhance microbial protein synthesis by supplying both starch and soluble protein, and ammonia-N. This was demonstrated by Kuoppala et al. (2021), who reported 38 and 28 g/d greater microbial N omasal flow in cows supplemented with faba bean compared with control or rapeseed meal supplemented cows, respectively. Even though greater rumen degradability can benefit microbial growth, these characteristics also result in lower overall N utilization of faba bean protein. To reduce the extent of rumen degradation, feed processing, such as dehulling, flaking, and heat treatment, can improve the nutritive value of faba bean (Pitkänen et al., 2023).

This short-term in vitro batch-culture study provides initial insights into utilization of faba bean protein in the rumen, but was limited in incubation time (10 h) as well as comparison to other protein feed sources. The results confirmed the high content of soluble true protein N in faba bean protein. This protein fraction was largely degraded to ammonia-N and partly incorporated into bacterial N within the ruminal incubation medium. Similarly, the INSN fraction was deaminized to some extent into ammonia-N and subsequently taken up by rumen bacterial-N pool, but also showed an immediate incorporation of part of INSN into bacterial N. The fiber-bound N of faba bean protein seemed to be unavailable for rumen microbes within a 10-h incubation when provided as the sole N source and was neither deaminized nor incorporated into bacterial N. Future studies, both in vitro and in vivo, are required to further evaluate especially the degradation rate and utilization of faba bean soluble protein in the rumen, as well as its contribution to MP supply.
